# Prenatal exposure to nitrate alters uterine morphology and gene expression in adult female F1 generation rats

**DOI:** 10.20945/2359-4292-2024-0085

**Published:** 2024-11-06

**Authors:** André Gilberto Cassiani, Thiago Pinheiro Arrais Aloia, Érica Kássia Sousa-Vidal, Sérgio Podgaec, Carla de Azevedo Piccinato, Caroline Serrano-Nascimento

**Affiliations:** 1 Hospital Israelita Albert Einstein São Paulo SP Brasil Hospital Israelita Albert Einstein, São Paulo, SP, Brasil; 2 Universidade de São Paulo Faculdade de Medicina de Ribeirão Preto Departamento de Ginecologia e Obstetrícia Ribeirão Preto SP Brasil Departamento de Ginecologia e Obstetrícia da Faculdade de Medicina de Ribeirão Preto, Universidade de São Paulo, Ribeirão Preto, SP, Brasil; 3 Universidade Federal de São Paulo São Paulo SP Brasil Universidade Federal de São Paulo, São Paulo, SP, Brasil

**Keywords:** DOHaD, nitrate, uterus, female rats

## Abstract

**Objective:**

Nitrate is ubiquitously found in the environment and is one of the main components of nitrogen fertilizers. Previous studies have shown that nitrate disrupts the reproductive system in aquatic animals, but no study has evaluated the impact of nitrate exposure on the uterus in mammals. This study aimed to evaluate the impact of maternal exposure to nitrate during the prenatal period on uterine morphology and gene expression in adult female F1 rats.

**Materials and methods:**

Pregnant Wistar rats were either treated with sodium nitrate 20 mg/L or 50 mg/L dissolved in drinking water from the first day of pregnancy until the birth of the offspring or were left untreated. On postnatal day 90, the uteri of female offspring rats were collected for histological and gene expression analyses. Morphometric analyses of the uterine photomicrographs were performed to determine the thickness of the layers of the uterine wall (endometrium, myometrium, and perimetrium) and the number of endometrial glands.

**Results:**

The highest nitrate dose increased the myometrial thickness of the exposed female rats. Treatment with both nitrate doses reduced the number of endometrial glands compared with no treatment. Additionally, nitrate treatment significantly increased the expression of estrogen receptors and reduced the expression of progesterone receptors in the uterus.

**Conclusion:**

Our results strongly suggest that prenatal exposure to nitrate programs gene expression and alters the uterine morphology in female F1 rats, potentially increasing their susceptibility to developing uterine diseases during adulthood.

## INTRODUCTION

Endocrine disruptors are natural or synthetic exogenous agents that interfere with the synthesis, secretion, transport, and action of hormones endogenously produced by the body (1,2). Several studies evaluating the impact and mechanisms of exposure to these disruptors on the endocrine system function have reported changes in neurobehavioral, growth, and stress responses; increased incidence of chronic diseases and some types of cancer; and alterations in the development of the reproductive system (3,4). Humans are persistently exposed to low concentrations of these contaminants throughout their lives (5-7). It is worth noting that even exposure to extremely low doses of endocrine disruptors also induces relevant responses in the body, especially during critical periods of development (8-10).

The prenatal and early postnatal periods are the most critical stages of development and have the greatest impact on tissue/organ programming (11). Thus, the environment to which pregnant women are exposed during pregnancy and lactation has significant effects on the development of the fetus and newborn, which also determines the extent of susceptibility to the development of diseases in adulthood (12).

The uterus is highly susceptible to the harmful effects of endocrine disruptors (3,13-16). A previous study has shown that rats chronically exposed to BPA have an increased incidence of cystic endometrial hyperplasia, cystic ovaries, and complex uterine pathologies, such as adenomyosis, leiomyomas, cystic hyperplasia, and endometrial polyps (17). Similarly, neonatal exposure to tributyltin (TBT) leads to thickened luminal epithelium, increased uterine fibrosis and tissue inflammation, and decreased number of endometrial glands in adult animals compared with controls (18).

The use of nitrogen fertilizers to enhance growth and accelerate food production has significantly increased the levels of nitrogen oxides and nitrates in the atmosphere and water sources (19). In humans, nitrates are readily absorbed in the upper gastrointestinal tract after ingestion and are distributed throughout the body, where they are converted into nitrite at specific sites (20-24). Previous studies in vertebrates and invertebrates have shown an association between nitrate contamination and changes in hormonal levels and between decreased reproductive capacity and impaired endocrine function (25,26). Harmful effects of nitrate exposure on the reproductive system, particularly on steroidogenesis and occurring due to the excessive production of nitrogen compounds, have been described (26).

Although nitrate is widely distributed in the environment, most studies on the harmful effects of nitrate on reproduction have been conducted in aquatic animals. These studies have reported a decrease in the number and weight of embryos, as well as a reduction in the reproductive capacity of offspring from animals exposed to nitrate (27,28). Only a few studies have focused on evaluating the effects of nitrate on the reproductive system in mammal models (29-32), while no studies have evaluated the uterine effects of nitrate in rodent models. Therefore, the present study aimed to investigate the impact of prenatal exposure to nitrate on uterine morphology and gene expression in adult female rat offspring.

## MATERIALS AND METHODS

### Experimental model and treatment protocol

Two-month-old adult virgin female Wistar rats (four per group) were housed in an acclimatized environment at 22 ± 2 ºC, with a relative humidity of 55 ± 10%, and an inverted light/dark cycle of 12 hours. Water and feed were provided *ad libitum*. After the adaptation period (approximately 10 days), the rats were mated, and the vaginal smear was evaluated daily to identify the first day of pregnancy. The rats were then weighed, separated into cages, and randomly divided into the following experimental groups: control (C) group, which received filtered water not supplemented with any treatment; nitrate 20 (N20) group, which received filtered water supplemented with NaNO_3_ 20 mg/L, and nitrate 50 (N50) group, which received filtered water supplemented with NaNO_3_ 50 mg/L. The treatment was administered only during pregnancy. Treatment doses were based on previous studies evaluating the effects of nitrate on health in humans and other animals (20,33,34). After the birth of the offspring, treatment was interrupted. Female offspring were maintained with normal rat chow and filtered water until postnatal day 90 when they were anesthetized and euthanized by decapitation (4-6 animals per experimental group). The estrous cycle on the day of euthanasia was determined by vaginal smear analysis. Notably, more than 90% of the female rats were at the metestrus or diestrus phase of the estrous cycle on the day of euthanasia.

### Sample collection and preservation

After euthanasia, the uteri of the female offspring rats were collected, and the uterine horns were divided for molecular and histological analyses. One of the horns was snap-frozen and stored in a freezer at −80 ºC until processing for molecular analyses. The other horn was stored in a Methacarn fixative solution for histological analysis.

### Sample preparation for histological analysis

Uterine fragments measuring 3 mm^3^ were fixed for 12 hours in Methacarn, then dehydrated in alcohol, cleared in xylene, and embedded in paraffin. The paraffin blocks were sectioned at a thickness of 5 μm. The slides were stained with hematoxylin and eosin and mounted using Entellan.

### Uterine wall thickness measurement and endometrial gland count

A Zeiss AxioVert. A1 microscope (Jena, Germany) and Zeiss Axiocam 503 Color camera (Jena, Germany) were used for acquiring images of the uteri. Photomicrographs of each slide were obtained using a 5x objective to ensure the visualization of the entire section. Thickness was measured in cross-sections of each uterus at six distinct points, corresponding to clock positions (1, 3, 5, 7, 9, and 11 o'clock). At each clock position, the total uterine thickness (myometrium plus endometrium plus perimetrium), myometrium, and endometrium were measured using the Zen Lite software (Jena, Germany). Uterine glands were manually counted in the endometrial layer of the uterus in histological sections from all experimental groups.

### RNA isolation, reverse transcription, and gene expression analysis

Uterine horns were homogenized in Trizol (Invitrogen Life Technologies, Carlsbad, CA, USA) using Polytron equipment. Total RNA was extracted according to the manufacturer's protocol. The RNA concentration in each sample was measured using a NanoDrop spectrophotometer. Subsequently, RNA was reverse transcribed into cDNA using M-MLV Reverse Transcriptase (Invitrogen Life Technologies). Real-time polymerase chain reaction (PCR) assays were performed using 200 nM of each primer pair designed for estrogen receptor alpha (*Esr1*), estrogen receptor beta (*Esr2*), and progesterone receptor (*Pgr*). The expression of *Rpl19* was used as an endogenous control in each reaction. Real-time PCR was performed using the QuantStudio 6 Real-time PCR System (Applied Biosystems, Thermo Fisher Scientific, Waltham, MA, USA). All reactions were performed in duplicate for each sample. Melting curves were analyzed to confirm the amplification of a single product in the PCR. For quantitative analyses, the results were calculated using the 2^-ΔΔCt^ method (35).

### Statistical analysis

Statistical analyses were performed using the software SPSS, version 24 (IBM Corp., Armonk, NY, USA), and GraphPad Prism, version 5.0 (GraphPad Software, Inc., La Jolla, CA, USA), considering a significance level of 5%. All numerical values are expressed as ratios of means or estimated mean ± standard deviation of the mean. Ratios of the means were calculated for comparisons with the C group.

Generalized estimating equation (GEE) models were used to measure uterine thickness accounting for correlations among measurements from different photographs of the same slide and from different locations within the same photograph. The Gamma distribution was applied, and a compound symmetry correlation structure was considered in all models (36).

The number of endometrial glands was compared among experimental groups using a GEE model (37), which accounted for correlations among different photographs of the same slide, with negative binomial distribution and logarithmic link function. The mean ratios of the treated groups (N20 and N50) were compared with those of the C group.

To analyze gene expression data, comparisons among the different experimental groups were performed using a one-way analysis of variance followed by the Student-Newman-Keuls test.

## RESULTS

### Effect of intrauterine nitrate exposure on body weight

The treatment of pregnant rats with sodium nitrate 20 mg/L or 50 mg/L did not significantly alter the duration of the gestational period or the number of offspring rats. However, nitrate-exposed F1 rats presented significantly reduced body weight compared with control animals. Notably, the rats’ body weight in adulthood was not significantly different between the nitrate-exposed and control groups (Table 1).

**Table 1:** Nitrate-induced effects on the duration of the gestational period, number of offspring rats, and body weight at birth and during adulthood

Groups	GPD (days)	NOF	BWB (g)	BWA (g)
C	21.0 ± 0.41	11 ± 0.63	5.470 ± 0.08	230 ± 3.1
N20	21.5 ± 0.28	12 ± 0.41	5.095 ± 0.08**	225 ± 3.0
N50	21.5 ± 0.50	11 ± 0.33	5.071 ± 0.07**	239 ± 7.1

Abbreviations: BWA, body weight during adulthood; BWB, body weight at birth; C, control group; GPD, gestational period duration; N20, nitrate 20 mg/L group; N50, nitrate 50 mg/L group; NOF, number of offspring rats. The results are expressed as mean ± standard error of the mean (SEM). N = 4-10 per group.

** P < 0.001 *versus* C.

### Effect of prenatal nitrate exposure on uterine wall thickness

**Figure 1:**
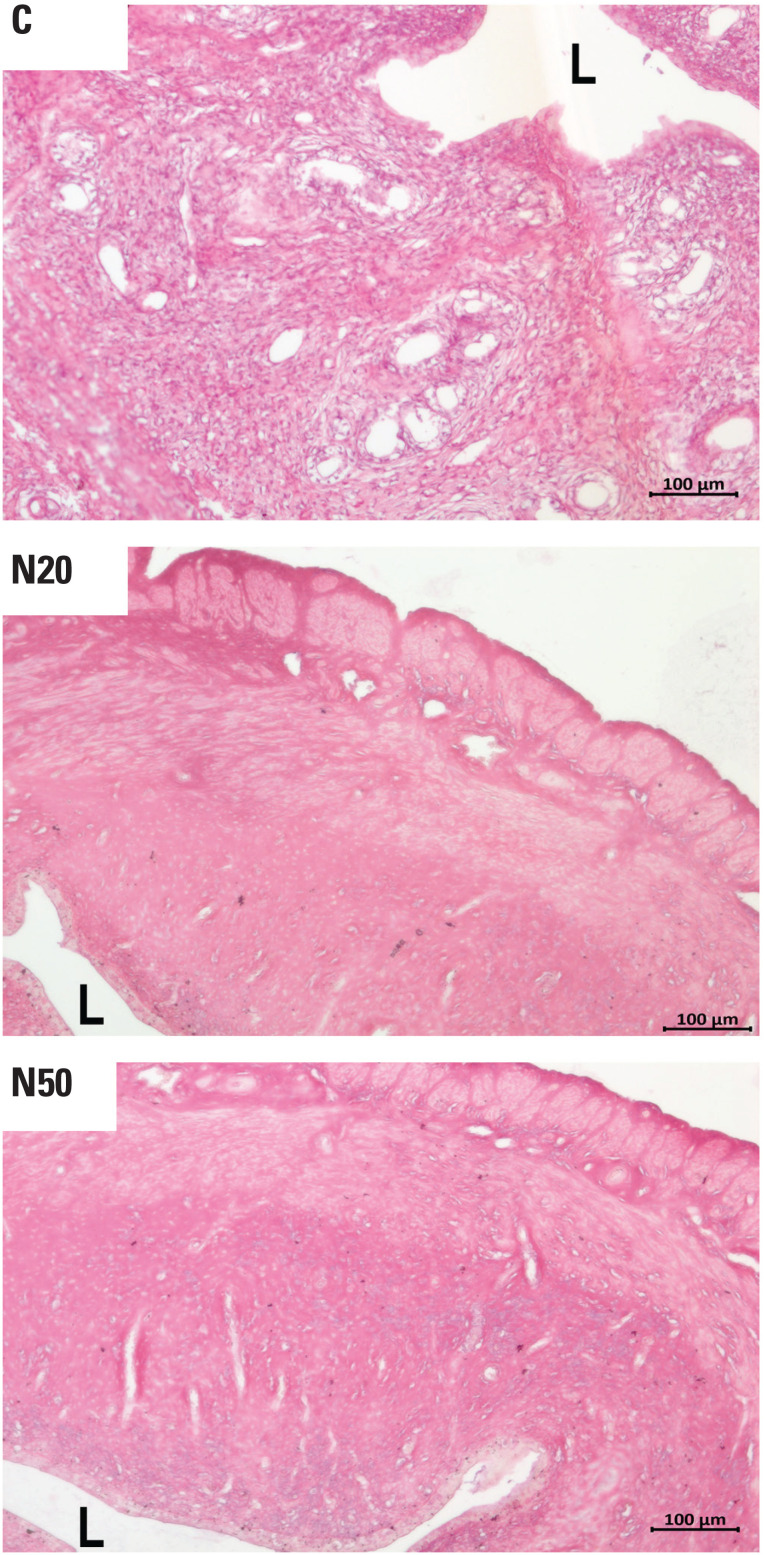
Histological sections of the uterine walls of adult female offspring rats exposed (N20 and N50) and not exposed (C) to nitrate during the prenatal period. Magnification: 100x. Hematoxylin and Eosin. Abbreviations: C, control group; L, uterine lumen; N20, nitrate 20 mg/L group; N50, nitrate 50 mg/L group.

**Figure 2:**
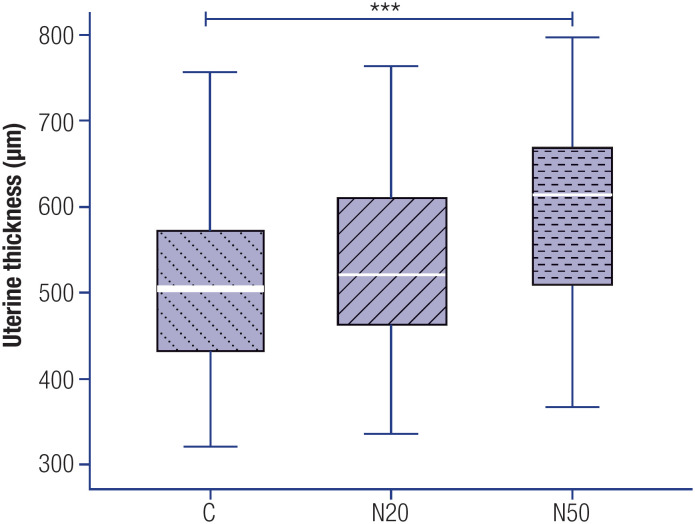
Uterine wall thickness in female rats exposed or not exposed to nitrate during the prenatal period. Photomicrographs of histological slides were used to measure uterine thickness, obtained with a 5x objective, ensuring the visualization of the entire section. All values are expressed in micrometers (μm). The white markers within the box plots represent the median values, while the whiskers indicate the range of the data. *** P < 0.001 compared with the C group. Abbreviations: C, control group; N20, nitrate 20 mg/L group; N50, nitrate 50 mg/L group.

Figure 1 illustrates the histological patterns of uterine walls in adult female offspring of animals from the C, N20, and N50 groups. Figure 2 demonstrates the mean thickness of the uterine wall in the animals exposed to nitrate and controls. The results indicated a 15.5% increase in the mean ratio of uterine thickness in the N50 group compared with the C group (p = 0.003). In contrast, no significant difference was observed between the mean ratios in the N20 and C groups, or between the N50 and N20 groups.

### Effect of prenatal nitrate exposure on myometrial and endometrial thickness

**Figure 3:**
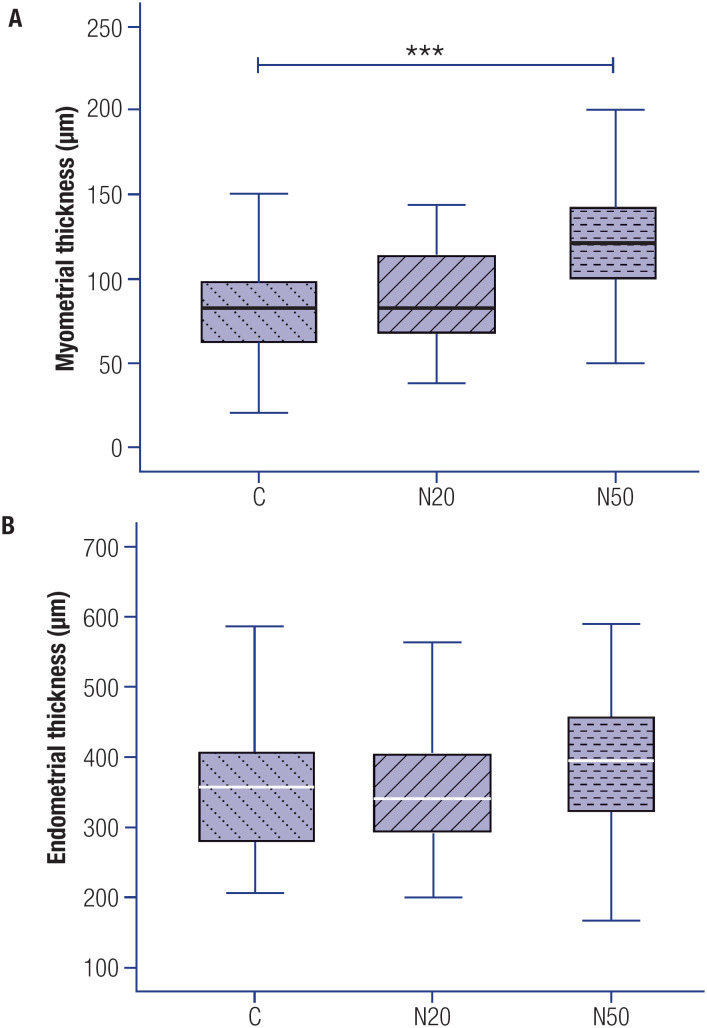
Myometrial and endometrial thickness of rats’ uteri exposed or not exposed to nitrate during the prenatal period. The measurement of myometrial (**A**) and endometrial (**B**) thickness was performed using photomicrographs of histological slides obtained with a 5x objective, ensuring the visualization of the entire section. All values are expressed in micrometers (μm). The white and black markers within the box plots represent the median values, while the whiskers indicate the range of the data. *** P < 0.001 compared with the C group. Abbreviations: C, control group; N20, nitrate 20 mg/L group; N50, nitrate 50 mg/L group.

The myometrial thickness was significantly increased (48.8%) in the N50 group compared with the C group (p < 0.001), but no significant differences were observed between the N20 and C groups or the N20 and N50 groups (Figure 3A). Endometrial thickness did not differ significantly between the C and N20 groups, the C and N50 groups, or the N20 and N50 groups (Figure 3B).

### Effect of prenatal nitrate exposure on the number of endometrial glands

**Figure 4:**
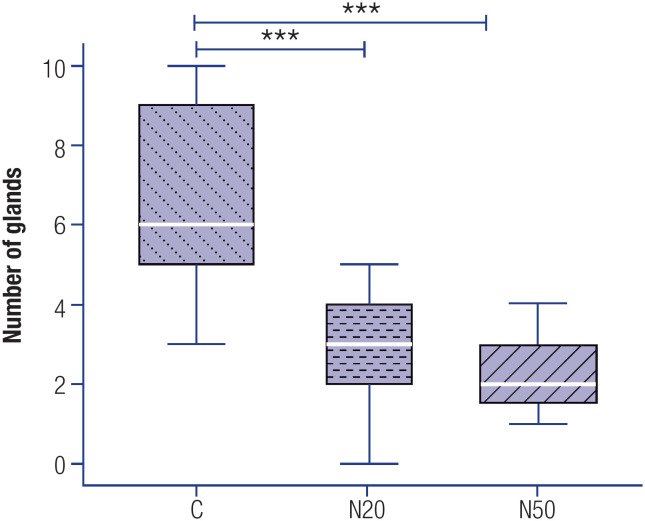
Number of endometrial glands in female rats exposed or not exposed to nitrate during the prenatal period. The number of endometrial glands was measured in photomicrographs obtained using a 5x objective. The white markers within the box plots represent the median values, while the whiskers indicate the range of the data. *** P < 0.001 compared with the C group. Abbreviations: C, control group; N20, nitrate 20 mg/L group; N50, nitrate 50 mg/L group.

Maternal exposure to nitrate during the prenatal period significantly reduced the number of endometrial glands in the uterus of the female offspring rats compared with the control animals (Figure 4). No differences in the number of glands were observed between the nitrate-exposed groups (N20 *versus* N50).

### Effect of prenatal nitrate exposure on the expression of sex steroid receptors in uteri of female offspring rats

**Figure 5:**
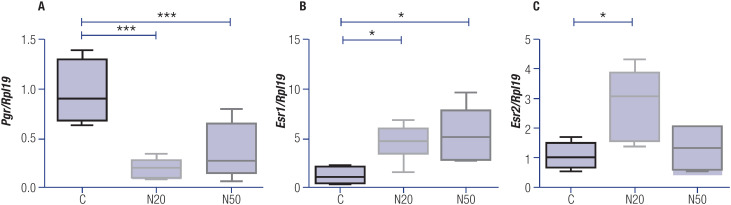
Gene expression of estrogen and progesterone receptors in uteri of rats exposed or not exposed to nitrate. The mRNA expression of (**A**) progesterone receptor (*Pgr*), (**B**) estrogen alpha receptor (*Esr1*), and (**C**) estrogen beta receptor (*Esr2*) was determined using real-time polymerase chain reaction (PCR), with *Rpl19* mRNA serving as the internal control. The data are presented as mean ± standard deviation in arbitrary units (AU) (n = 4-6). The black markers within the box plots represent the mean values, while the whiskers indicate the range of thse data. * P < 0.05; *** p < 0.001 *versus* the C group. Abbreviations: C, control group; N20, nitrate 20 mg/L group; N50, nitrate 50 mg/L group.

Maternal exposure to nitrate during the prenatal period altered the mRNA expression of estrogen and progesterone receptors in the uteri of adult female F1 rats. Both doses of nitrate significantly reduced uterine *Pgr* expression in relation to the control group (Figure 5A). No significant difference in *Pgr* expression was observed between the N20 and N50 groups.

The mRNA expression of *Esr1* (Figure 5B) and *Esr2* (Figure 5C) increased significantly in the uteri of the female offspring rats exposed to the lowest dose of nitrate (N20) during the prenatal period. Notably, exposure to the highest dose of nitrate (N50) increased mRNA expression of *Esr1* but did not alter the mRNA expression of *Esr2* compared with the control (C) group. There were no significant differences in expression of estrogen receptors between the nitrate-treated groups.

## DISCUSSION

The reproductive system is particularly vulnerable to the harmful effects of endocrine disruptors (38). Many studies have reported unfavorable reproductive outcomes in both animals and humans exposed to endocrine disruptors (3,4,39). The results from the present study confirm the vulnerability of the reproductive system, strongly suggesting that prenatal exposure to nitrate changes the uterine morphology of female offspring rats, affecting the expression of key genes responsible for uterine development and function.

Although nitrate – a known endocrine disruptor – is widely distributed in the environment, few studies have focused on evaluating its effects on the reproductive system, specifically on the uterus of animal models. Studies on the harmful effects of nitrate on mammals are also lacking (27-29), thus emphasizing the novelty of our study. Morphological analysis of the uterus of F1 females showed that nitrate exposure, especially at the highest treatment dose (50 mg/L, N50 group), significantly increased the total thickness of the uterine wall and the thickness of the myometrium compared with no nitrate exposure (C group). In contrast, no significant changes in endometrial thickness were observed. Notably, previous studies have not observed changes in uterine weight in two different generations of female rats exposed to yttrium nitrate or lanthanum nitrate (40,41).

Few studies have evaluated the impact of nitrate exposure on the mammalian reproductive system, particularly during critical windows of susceptibility. In this context, a recent study investigated the two-generation effects of yttrium nitrate exposure in Sprague-Dawley rats, using doses of 0 mg/kg, 10 mg/kg, 30 mg/kg, and 90 mg/kg. The female F1 generation rats exposed to the low-dose treatment presented reduced weight gain compared with the control group (40). Another study from the same group, also using Sprague-Dawley rats, exposed the animals to lanthanum nitrate and observed no changes in food intake or body weight in F1 or F2 generation rats (41). Our study also found no differences in body weight among nitrate-exposed animals. These discrepant results may be due to the use of different nitrate compounds and to the fact that, unlike in previous studies, the treatment we implemented in ours was limited to the prenatal period of the F0 generation in our experimental model. Studies on perinatal exposure to other endocrine disruptors, such as tributyltin (TBT) and BPA, have demonstrated important uterine morphological and functional changes in female offspring, including an increased incidence of cystic endometrial hyperplasia and development of luminal epithelium thickening in adult rats (17,18), which is similar to the observations in the present study. Thus, the increased thickness of uterine layers observed after prenatal exposure to nitrate may be due to the aberrant proliferation of uterine cells, as previously suggested for other disruptors (17,42). High proliferative activity of uterine cells has been associated with increased susceptibility to the development of benign or malignant uterine lesions. The aberrant proliferation of uterine layer cells and endometrial glands is considered the first stage in the development of uterine diseases related to cell hyperplasia (43).

A reduced number of endometrial glands was observed in the uterus of nitrate-exposed rats at both treatment doses. This finding reinforces the hypothesis that exposure to endocrine disruptors during critical development periods may result in alterations in the function and morphology of different tissues and organs (44). Thus, this result indicates another important harmful effect of nitrate in the reproductive system of rats, as the reduction in the number of endometrial glands and nutritive secretions potentially compromises the implantation and initial maintenance of embryos (45).

Future studies are necessary to elucidate the molecular mechanisms underlying morphological changes induced by gestational exposure to nitrate. However, the role of ovarian steroids in maintaining uterine morphology and function is irrefutable (46). Thus, the results regarding estrogen and progesterone receptor expression suggest that the imbalanced hormonal action in the uterus, induced by intrauterine exposure to nitrate, is related to the observed morphological changes. Signaling disturbances triggered by the hormone-receptor interactions contribute to the development of uterine dysfunctions. Therefore, the altered mRNA expression of *Pgr, Esr1,* and *Esr2* in nitrate-exposed animals potentially suggests a relationship between impaired hormone signaling and the observed changes in uterine histoarchitecture.

The stimulatory effect of estrogen on uterine cells is largely unclear. In rodents, in addition to its endometrial effects, estrogen induces epithelial cell hypertrophy and hyperplasia and stimulates cell proliferation in the myometrial layers (47-52). The results of the present study indicate that the myometrium was the uterine layer that underwent the most significant morphological changes in nitrate-exposed animals. The hyperplastic appearance of the uterus in these nitrate-exposed animals could be related to increased mRNA expression of *Esr1* and *Esr2* and reduced mRNA expression of *Pgr*. Indeed, the reduced number of endometrial glands may also be related to reduced uterine mRNA expression of *Pgr* in nitrate-exposed female rats. These findings are consistent with previous studies describing the harmful effects of intrauterine exposure to 2,3,7,8-tetrachlorodibenzo-p-dioxin (TCDD), an endocrine disruptor that induces uterine dysfunctions similar to those of endometriosis in progeny animals, persisting up to the third generation (53). These effects result from reduced uterine responsiveness to progesterone and increased expression of ESR2 in uterine stromal and epithelial cells (53,54).

The lack of serum estrogen and progesterone level measurements in our experimental model is a limitation of our study. However, other important regulators of the expression of estrogen receptor and progesterone receptor genes (55), beyond estrogen and progesterone, may also be involved. Additionally, nitrate itself could directly dysregulate the expression of estrogen receptor and progesterone receptor genes. Preliminary data from our group indicate that nitrate exposure during the intrauterine period alters the function of the hypothalamic-pituitary-thyroid axis in these animals. Thyroid hormones are known to regulate the expression of sex steroid hormone receptors and interfere with the reproductive function of mammals (56,57). Thus, the mechanism through which the expression of estrogen receptor and progesterone receptor genes was altered in the nitrate-treated groups could be explained by alterations in estradiol or progesterone levels but also by alterations in other hormones, or even by a direct effect of nitrate on these receptors’ expressions during the prenatal period. Since several mechanisms could be involved in the dysregulated expression of the estrogen receptor and progesterone receptor genes, a comprehensive study of potential mechanisms explaining this alteration should be further explored.

The limitations of this study include the fact that we did not weigh the rats’ uteri or follow up to observe the reproductive performance or development of pathologies in the female F1 rats and subsequent generations. We also did not evaluate the impact of nitrate exposure on the estrous cycle periodicity in the female F1 rats. Despite these limitations, our study is the first to show the deleterious impact of prenatal nitrate exposure on uterine histoarchitecture and gene expression in female rats, using nitrate doses previously considered safe for humans.

In conclusion, the present study is the first to demonstrate that intrauterine exposure to nitrate negatively affects uterine morphology and gene expression in adult female F1 rats. The exposure to this ubiquitous environmental endocrine disruptor increased uterine thickness, reduced the number of endometrial glands, and altered the expression of *Pgr, Esr1,* and *Esr2* in the uterus. The results presented herein confirm that the prenatal period is a critical stage of vulnerability to the harmful effects of endocrine disruptors.
